# Accidental exposures to peanut in a large cohort of Canadian children with peanut allergy

**DOI:** 10.1186/s13601-015-0055-x

**Published:** 2015-04-02

**Authors:** Sabrine Cherkaoui, Moshe Ben-Shoshan, Reza Alizadehfar, Yuka Asai, Edmond Chan, Stephen Cheuk, Greg Shand, Yvan St-Pierre, Laurie Harada, Mary Allen, Ann Clarke

**Affiliations:** Division of Allergy and Clinical Immunology, Department of Medicine, University of Montreal, Montreal, QC Canada; Division of Pediatric Allergy and Clinical Immunology, Department of Pediatrics, McGill University Health Centre, Montreal, QC Canada; Division of Allergy and Clinical Immunology, Department of Pediatrics, McGill University Health Centre, Montreal, QC Canada; Division of Dermatology, Department of Medicine, Queen’s University, Kingston, ON Canada; Division of Allergy and Immunology, Department of Pediatrics, British Columbia Children’s Hospital, University of British Columbia, Vancouver, BC Canada; Allergist/Clinical Immunologist, Calgary, AB Canada; Division of Clinical Epidemiology, Department of Medicine, McGill University Health Centre, Montreal, QC Canada; Division of Clinical Epidemiology, Department of Medicine, McGill University Health Centre, Montreal, QC Canada; Anaphylaxis Canada, Toronto, ON Canada; Allergy/Asthma Information Association, Toronto, ON Canada; Division of Rheumatology, Department of Medicine, University of Calgary, Calgary, AB Canada

**Keywords:** Peanut allergy, Accidental exposure, Epidemiology, Food allergy, Treatment

## Abstract

**Background:**

We previously estimated that the annual rate of accidental exposure to peanut in 1411 children with peanut allergy, followed for 2227 patient-years, was 11.9% (95% CI, 10.6, 13.5). This cohort has increased to 1941 children, contributing 4589 patient-years, and we determined the annual incidence of accidental exposure, described the severity, management, location, and identified associated factors.

**Findings:**

Children with physician-confirmed peanut allergy were recruited from Canadian allergy clinics and allergy advocacy organizations from 2004 to May 2014. Parents completed questionnaires regarding accidental exposure to peanut over the preceding year. Five hundred and sixty-seven accidental exposures occurred in 429 children over 4589 patient-years, yielding an annual incidence rate of 12.4% (95% CI, 11.4, 13.4). Of 377 accidental exposures that were moderate or severe, only 109 (28.9%) sought medical attention and of these 109, only 40 (36.7%) received epinephrine. Of the 181 moderate/severe accidental exposures treated outside a health care facility, only 11.6% received epinephrine. Thirty-seven percent of accidental exposures occurred at home. In multivariate analyses, longer disease duration, recruitment through an allergy advocacy association, and having other food allergies decreased the likelihood of accidental exposures. Age ≥ 13 years at study entry and living with a single parent increased the risk.

**Conclusion:**

Despite increased awareness, accidental exposures continue to occur, mainly at home, and most are managed inappropriately by both health care professionals and caregivers. Consequently, more education is required on the importance of strict allergen avoidance and the need for prompt and correct management of anaphylaxis.

**Electronic supplementary material:**

The online version of this article (doi:10.1186/s13601-015-0055-x) contains supplementary material, which is available to authorized users.

## Introduction

Research conducted over the past 25 years has estimated that the annual rate of accidental exposure (AE) in children with peanut and/or nut allergy ranges between 3 and 50% [1-6]. We previously conducted the largest longitudinal study on the rate and predictors of AE in children with peanut allergy and observed that in a Canadian cohort of 1411 children with peanut allergy, recruited between 2004 and 2009, and followed for 2227 patient-years, the annual rate of AE was 11.9% (95% confidence interval, CI, 10.6, 13.5) [7]. The cohort has increased to 1941 children, providing 4589 patient-years of follow-up, and in this manuscript, we determined the annual incidence of AE in this expanded cohort and described the severity, management, and location of the AEs, and identified factors associated with AE.

## Methods

### Study design

Children with physician-confirmed peanut allergy (eligibility criteria below) were recruited from the Allergy Clinics at the Montreal Children’s Hospital (MCH) and British Columbia’s Children’s Hospital (BCCH) and Canadian food allergy advocacy organizations; recruitment began at the MCH in 2004, at BCCH in 2013, and from the associations in 2006 and continued from all sources through to May 2014. Details on the cohort have been published elsewhere [7-10]. Patients were mailed a questionnaire at study entry (Additional file 1) and every two years (Additional file 2); starting in 2010, follow-up questionnaires were administered annually (Additional file 2). Details collected on AEs included the food ingested and the signs, symptoms, location, and treatment. Parents also reported on demographics, the allergic child’s history of atopy, and the child’s initial reaction to peanut.

The study was approved by the McGill University Health Centre and BCCH Research Ethics Board.

### Criteria for diagnosis of peanut allergy

Children were considered allergic to peanut if they had:

- A convincing history [6,11] of an allergic reaction and a positive skin prick test (SPT) ≥ 3 mm to peanut or peanut-specific IgE ≥ 0.35 kU/l [12-14] or
- An uncertain history of an allergic reaction or no previous exposure and either a positive SPT ≥ 3 mm AND peanut-specific IgE ≥ 15 kU/L [15,16] OR a positive challenge to peanut.

A convincing clinical history of peanut allergy was defined as a minimum of two mild signs or symptoms or either one moderate or one severe sign or symptom that was likely IgE mediated and occurred within 120 min after peanut ingestion or contact. Reactions were considered mild if they involved only pruritus, urticaria, flushing, or rhinoconjunctivitis; moderate if angioedema, throat tightness, gastrointestinal complaints, or breathing difficulties (other than wheeze); and severe if wheeze, cyanosis, or circulatory collapse [6,11].

### Statistical analysis

Descriptive statistics were compiled for all variables. The annual incidence rate of AE was expressed as the number of events divided by the sum of the patient-years at risk. As a sensitivity analysis, the rate of AE was also calculated by: 1) excluding those with one mild symptom or no previous exposure and positive confirmatory tests and 2) including only those with a positive food challenge, history of anaphylaxis [17], or convincing history with a SPT ≥ 8 mm or a peanut-specific IgE ≥ 15 kU/L [15].

Univariate and multivariate Cox regression analyses were used to examine potential predictors of the hazard of an AE. Predictors were selected using backward stepwise selection. Potential predictors for the Cox regression included sex, ethnicity, age at study entry, disease duration, source of recruitment (i.e., food allergy advocacy associations versus allergy clinics), other atopic conditions, severity of initial reaction to peanut, whether the child attended a school prohibiting peanut, and parental factors (i.e., living arrangement, age, level of education and employment). All statistical analyses were done with Stata, version 13 (StataCorp LP, Texas).

## Findings

### Patient characteristics

Of 2759 patients surveyed, 1941 (70.4%) responded with 35.9% of respondents recruited from the allergy advocacy associations. Patients were predominantly male (62.1%) and Caucasian (88.0%). The mean age (standard deviation, SD) and disease duration at the time of the initial questionnaire was 6.9 (4.0) and 4.7 (4.0) years, respectively, and the mean length of follow up was 2.4 years (SD 1.4) (Table 1).

**Table 1:** **Comparing demographic and clinical characteristics of participants with and without accidental exposures**

	**With AE n = 429**	**Without AE n = 1512**	**Difference (95% CI)**
Male, %	62.0	62.2	−0.2 (−5.4, 5.0)
Ethnicity, % Caucasian	86.5	88.5	−2.0 (−5.6, 1.6)
Age at diagnosis,* years, mean (SD)	2.3 (2.1)	2.1 (1.7)	0.2 (0, 0.4)
Age at initial questionnaire, years, mean (SD)	6.2 (3.9)	7.1 (4.0)	−0.9 (−1.3, −0.4)
Disease duration at initial questionnaire, years, mean (SD)	3.9 (3.7)	5.0 (4.0)	−1.1 (−1.5, −0.7)
Observation interval, years, mean (SD)	2.6 (1.4)	2.3 (1.4)	0.3 (0.2, 0.4)
Age ≥ 13 years at initial questionnaire, %	8.9	9.9	−1.0 (−4.1, 2.1)
Recruited through allergy associations, %	32.2	37.0	−4.8 (−9.8, 0.2)
Personal history of eczema, %	51.3	51.7	−0.4 (−5.7, 5.0)
Personal history of asthma, %	45.7	49.9	−4.2 (−9.5, 1.2)
Personal history of rhinitis, %	33.6	35.6	−2.0 (−7.1, 3.1)
Personal history of other food allergy, %	45.9	51.9	−6.0 (−11.3, −0.7)
Initial reaction moderate/severe, **%	58.9	69.1	−10.2 (−15.4, −5.0)
Initial reaction severe, %	11.2	14.5	−3.3 (−6.8, 0.2)
Attending a school prohibiting peanut, %	80.3	80.2	0.1 (−4.4, 4.6)
Single parent household, %	8.4	6.6	1.8 (−1.2, 4.8)
Age of parents, years, mean (SD)	38.6 (5.8)	39.2 (5.7)	−0.7 (−1.3, −0.1)
Mother’s education and work status, %			
Post-secondary education	87.4	88.7	−1.3 (−4.8, 2.3)
Completed university	60.7	61.0	−0.3 (−5.5, 5.0)
Currently employed	68.7	70.1	−1.5 (−6.6, 3.7)
Father’s education and work status, %			
Post-secondary education	78.0	79.5	−1.5 (−6.0, 3.0)
Completed university	51.6	53.8	−2.2 (−7.6, 3.3)
Currently employed	88.8	91.6	−2.8 (−6.2, 0.7)

*The age of diagnosis of peanut allergy was the earliest of the age of the first reaction or confirmatory testing.

**Mild signs/symptoms: pruritus, urticaria, flushing, rhinoconjunctivitis; moderate: angioedema, throat tightness, gastrointestinal complaints, breathing difficulties other than wheeze; severe: wheeze, cyanosis, circulatory collapse [6,11].

Participants experiencing an AE were younger and had a shorter disease duration at the time of the initial questionnaire, had a longer observation interval, were less likely to have other food allergies and to have an initial reaction that was moderate or severe, and had slightly younger parents (Table 1).

Overall, 69 (3.6%) participants were defined as having peanut allergy based on a positive oral food challenge, 1698 (87.5%) had a convincing clinical history and positive confirmatory testing, and 64 (3.3%) had one mild symptom and positive confirmatory testing and 110 (5.7%) had no clinical reaction and positive confirmatory testing.

### Risk, severity, management, and location of accidental exposures

**Figure 1:**
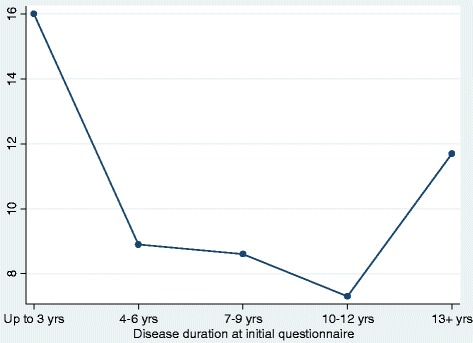
**Annual incidence rate of accidental exposure stratified by disease duration.**

Five hundred and sixty-seven AEs occurred in 429 children over 4589 patient-years, yielding an annual incidence rate of 12.4% (95% CI, 11.4, 13.4). Figure 1 depicts these AEs stratified by disease duration. The rate was similar when those with one mild symptom or no previous exposure and positive confirmatory tests were excluded (n remaining = 1767; rate of AE: 13.3%, 95% CI, 12.2, 14.4) and when only those with a positive food challenge, history of anaphylaxis, or a convincing history and a SPT ≥ 8 mm or a peanut-specific IgE ≥ 15 kU/L were included (n = 1541; rate of AE: 13.5% CI 12.4, 14.8).

Among 567 AEs, 149 (26.3%) of the corresponding initial reactions to peanut were mild, 284 (50.1%) were moderate, and 64 (11.3%) severe; for 70 (12.3%) AEs, there was no previous peanut exposure (and these patients were diagnosed based on confirmatory testing) or the reaction severity was unknown. Among 463 AEs preceded by an initial reaction (with known severity), 26.3% of AEs were more severe than the initial reaction, 21.0% were less severe, and 52.7% were of comparable severity.

No treatment was administered for 36.5% of the 148 mild AEs, 25.6% of the 292 moderate AEs, and 14.1% of the 85 severe AEs (the reaction severity was unknown for 42 AE, 4 of these received no treatment, and for 37 of these the treatment was unknown). Of 292 AEs that were moderate, only 73 (25.0%) sought medical attention and of these 73, only 22 (30.1%) received epinephrine. Of the 144 moderate AEs treated outside a health care facility, only 13 (9.0%) received epinephrine (the remaining 75 moderate AEs received no treatment). Of the 85 AEs that were severe, only 36 (42.4%) sought medical attention and of these 36, 18 (50.0%) received epinephrine. Of the 37 severe AEs treated outside a health care facility, only 8 (21.6%) received epinephrine (the remaining 12 severe AEs received no treatment).

Thirty-seven percent of AEs occurred at home, 14.3% at relatives’/friends’ homes, 9.3% in restaurants, 4.9% at schools/day-cares prohibiting peanut, 3.0% at schools/day-cares allowing peanuts, and 31.6% at other or unknown places.

### Predictors of accidental exposure

Longer disease duration (adjusted hazard ratio (HR): 0.90, 95% CI: 0.88, 0.93), recruitment from food allergy advocacy associations (HR: 0.75, 95% CI: 0.63, 0.91), and having other food allergies (HR: 0.81, 95% CI: 0.68, 0.96) decreased the likelihood of an AE (Table 2). Age ≥ 13 years at study entry (HR: 2.22, 95% CI: 1.44, 3.41) and residing in a single parent household (HR: 1.55, 95% CI: 1.14, 2.10) increased the risk of AE.

**Table 2:** Univariate and multivariate predictors* of accidental exposures**

	**Univariate**	**Most informative multivariate**
	**HR (95% CI)**	**HR (95% CI)**
Caucasian	0.70 (0.54, 0.90)	Not Included
Age at study entry	0.93 (0.91, 0.96)	Not Included
Disease duration at entry	0.92 (0.89, 0.94)	0.90 (0.88, 0.93)
Age ≥ 13 years at study entry	Non Significant	2.22 (1.44, 3.41)
Recruited through allergy associations	0.65 (0.54, 0.77)	0.75 (0.63, 0.91)
Personal history of rhinitis	0.82 (0.69, 0.97)	Not Included
Personal history of other food allergy	0.75 (0.64, 0.89)	0.81 (0.68, 0.96)
Single parent household	1.46 (1.08, 1.98)	1.55 (1.14, 2.10)
Age of parents	0.97 (0.95, 0.98)	Not Included
Father’s education and work status		
Currently employed	0.72 (0.55, 0.94)	Not Included

HR: Hazard ratio.

*Only significant predictors are indicated.

**Potential predictors for the Cox regression included sex, ethnicity, age at study entry (i.e., at the time the patient starts to be observed, which could be up to one year prior to the initial questionnaire), disease duration, source of recruitment (i.e., food allergy advocacy associations versus allergy clinics), other atopic conditions, severity of initial reaction to peanut, whether the child attended a school prohibiting peanut, and parental factors (i.e., living arrangement, age, level of education and employment).

## Discussion

We have conducted the largest longitudinal study on the rate, treatment, and predictors of AE in children with peanut allergy. In our cohort of 1941 children, 567 AEs occurred in 429 children over 4589 patient-years, yielding an annual incidence rate of 12.4% (95% CI, 11.4, 13.4). Of 377 AEs that were moderate or severe, only 109 (28.9%) sought medical attention and of these, only 40 (36.7%) received epinephrine. In multivariate analyses, longer disease duration, recruitment through an allergy advocacy association, and having other food allergies decreased the likelihood of AEs, whereas age ≥ 13 years at study entry and living with a single parent increased the risk.

Longer disease duration is likely associated with a lower risk of AE because participants develop better allergen avoidance strategies over time. Participants who were adolescents at study entry are at higher risk, given equal disease duration, presumably due to their risk-taking behaviours [18]. Combining the independent effect of disease duration and age therefore explains the U-shaped relationship between disease duration and incidence of AE depicted in Figure 1. Participants with other food allergies likely perceive themselves at higher risk of having a severe AE and exercise more caution. Children recruited through food allergy advocacy associations were also less likely to experience an AE, possibly reflecting their enhanced awareness. As it is possible that parents of children recruited from hospital allergy clinics were also members of allergy associations (but we did not inquire about this), our estimate of the lower risk associated with membership is likely conservative.

Our annual incidence rate of 12.4% is substantially lower than the 50% and 33% reported in studies conducted in 1989 [5] and 2000 [4], respectively. This likely reflects enhanced societal awareness regarding the diagnosis, risks, and management of peanut allergy. However, our estimate exceeds that reported in recent studies. Clark et al. reported a rate of only 3.1% in 2008, but these participants with peanut and/or nut allergy received a comprehensive management program [1,2]. Neuman-Sunshine et al. observed a rate of 7.3% in a cohort of 782 patients where AEs were possibly underestimated as they were ascertained by chart review and a substantial portion may not have been allergic (37.9% were diagnosed based solely on an elevated peanut-specific IgE without any reaction) [3]. A high rate of AE has also been observed for other food allergies; Boyano-Martinez et al. reported that 40% of 88 children with allergy to cow’s milk and 21% of 92 children with allergy to hen’s egg reported an AE in the preceding year [19,20].

The low rate of usage of epinephrine is consistent with other studies [21-24], which report administration of epinephrine in less then 50% of cases where it is indicated.

We did not observe a difference in the percentage of AEs occurring in schools/daycares prohibiting (4.9%, 95% CI, 3.3, 7.1) versus allowing peanuts (3.0%, 95% CI, 1.8, 4.8). Failure to observe such a decreased rate in facilities restricting peanut may be due to increased awareness and enhanced vigilance among parents, school personnel, and children in schools permitting peanut. Further, peanut-free policies may create a false sense of security and foods brought to such facilities may inadvertently contain peanut and children who are allergic may believe that it is safe to share foods as they believe they are guaranteed to be peanut free.

Our study is limited in that inclusion of children with one mild symptom or no previous exposure and a peanut-specific IgE ≥ 15 kU/L but having an SPT between 3 and 7 mm may not have been sufficiently rigorous. However, the annual rate of AE did not differ between those children with one mild symptom or no previous exposure with a peanut-specific IgE ≥ 15 kU/L and SPT between 3 and 7 (n = 46, rate of AE: 3.3%, 95% CI, 0.9, 8.5) and SPT ≥ 8 mm (n = 128, rate of AE: 3.8%, 95% CI, 2.0, 6.7). For those 226 children with a convincing history with an SPT between 3 and 7 mm, the point estimate of the annual rate of AE was actually 10.8% (95% CI, 7.9, 14.5). It is possible the lower rate of AE observed in the 174 without a convincing history (9.0% of the entire cohort of 1941) suggests that some of these children were only sensitized. Alternatively, they may have had a higher threshold and required a larger amount of peanut to provoke an allergic reaction. Excluding these 174 children does not result in a clinically meaningful increase in the rate of AE (n = 1767, rate of AE: 13.3%, 95% CI, 12.2, 14.4) and as these children are still experiencing some AEs, omitting them from the analysis may inappropriately inflate our AE estimates.

Despite increasing efforts to provide information on the management of food allergy, AEs continue to occur, mainly in the child’s home, but also in peanut free schools/day-cares. Most moderate/severe AEs are managed inappropriately by caregivers and physicians. Consequently, more education is required on the importance of strict allergen avoidance and the need for prompt and correct management of anaphylaxis.

## Additional files

Additional file 1:
**Peanut allergy registry.** Baseline questionnaire sent to patients when first joining the peanut allergy registry. Additional file 2:
**Peanut allergy registry – follow up.** Follow up questionnaire sent to patients participating in the peanut allergy registry.

## Abbreviations

AE: Accidental exposure
BCCH: British Columbia Children’s Hospital
CI: Confidence interval
MCH: Montreal Children’s Hospital
HR: Hazards ratio
SD: Standard deviation
SPT: Skin prick test
