# Supplementary material for: Accidental exposures to peanut in a large cohort of Canadian children with peanut allergy
Source: Clin Transl Allergy. 2015 Apr 2;5:16. doi: 10.1186/s13601-015-0055-x (PMC4389801; doi:10.1186/s13601-015-0055-x)
Supplement: Additional file 1: — Peanut allergy registry. Baseline questionnaire sent to patients when first joining the peanut allergy registry. [file 13601_2015_55_MOESM1_ESM.pdf]

Peanut Allergy Registry

Date: (dd/mmm/yyyy)___ ___ _____________ _______ **Study ID #:** ☐ ☐☐☐☐

TO BE COMPLETED BY PARENT/GUARDIAN OF PEANUT ALLERGIC PARTICIPANT

1) What is the gender of your child? ☐Male ☐ Female

2) What is your child's date of birth? **(dd/mm/yyyy)** ________________________________

3) Has your child **ever** been diagnosed by a physician as having one of the following conditions?

☐ Asthma ☐Eczema ☐Hay fever or allergic rhinitis (stuffy/runny nose or frequent sneezing)

☐ Hives **from an unknown cause** (itchy skin rash that comes and goes over minutes to hours)

☐ Anaphylactic reaction (a severe, sudden, and life-threatening **allergic** reaction to a foreign substance that causes problems in multiple systems of the body. e.g.: wheezing or other breathing difficulties, total body hives, vomiting, loss of consciousness) **Please** specify the cause of anaphylaxis if known: ___________________________

☐ Food allergy (list all the foods) ____

4) Please check all that apply regarding the following conditions:

Asthma

Child's mother	☐ Yes	☐ No	☐ Resolved	☐ Don't know
Child's father	☐ Yes	☐ No	☐ Resolved	☐ Don't know
Child's sibling (s)	☐ Yes	☐ No	☐ Resolved	☐ Don't know

Eczema

Child's mother	☐ Yes	☐ No	☐ Resolved	☐ Don't know
Child's father	☐ Yes	☐ No	☐ Resolved	☐ Don't know
Child's sibling (s)	☐ Yes	☐ No	☐ Resolved	☐ Don't know

Hay fever or allergic rhinitis (stuffy nose or frequent sneezing)

Child's mother	☐ Yes	☐ No	☐ Resolved	☐ Don't know
Child's father	☐ Yes	☐ No	☐ Resolved	☐ Don't know
Child's sibling (s)	☐ Yes	☐ No	☐ Resolved	☐ Don't know

Hives (itchy skin rash that comes and goes over minutes to hours)

Child's mother	☐ Yes	☐ No	☐ Resolved	☐ Don't know
Child's father	☐ Yes	☐ No	☐ Resolved	☐ Don't know
Child's sibling (s)	☐ Yes	☐ No	☐ Resolved	☐ Don't know

Anaphylaxis (wheezing or other breathing difficulties, total body hives, vomiting or loss of consciousness from an allergic reaction)

Child's mother	☐ Yes	☐ No	☐ Resolved	☐ Don't know
Child's father	☐ Yes	☐ No	☐ Resolved	☐ Don't know
Child's sibling (s)	☐ Yes	☐ No	☐ Resolved	☐ Don't know

Food allergy
☐ Child's mother: list food (s) ____
☐ Child's father: list food (s)____
☐ Child's sibling (s): list food (s)____
☐ None of the above

5) Which epinephrine auto-injector does your child **currently have**?

☐ Epipen® ☐ Twinject™ ☐ Allerject™ ☐ Other, please specify: __________________

☐ I don't know ☐ My child does not have an Auto-Injector

6) At what age were **peanut/peanut-containing food(s)** introduced into the child's diet? ___________

If you do not remember when they were introduced please put your best guess: _________________

☐ I have not introduced peanut/peanut-containing food(s) into the diet

7) When was your child diagnosed by a physician as having **peanut** allergy? (mm/yyyy) ________ / _______

8) Has your child ever had any allergic reaction(s) to **peanut?** ☐ Yes (Please go to Question **9**)
☐ No (Please go to Question **12**)

9) FIRST REACTION TO PEANUT IN HIS/HER LIFE

Date of reaction (Approximate month/year) ____________________________________

A. Where did the reaction occur?

☐ Home ☐ Daycare-peanut allowed ☐ Daycare-peanut not allowed
☐ Restaurant ☐ School-peanut allowed ☐ School-peanut not allowed
☐ I don't know ☐Other _
____

B. How did the reaction occur? ☐ After ingestion ☐ After skin contact ☐ I don't know
☐ After inhalation ☐ Not determined

C. Was the epinephrine auto-injector (Epipen®, Twinject™, Allerject™) available?
☐ Yes ☐ No ☐ I don't know

D. If an epinephrine auto-injector was used, was it his/her own? ☐ Yes ☐ No ☐ I don't know
If No, please specify whose epinephrine auto-injector was used: ______________________________

E. Please check the symptoms that occurred:

☐ Hives ☐ Difficulty breathing ☐ Runny nose and watery eyes ☐ I don't know
☐ Redness ☐ Vomiting ☐ Passing out
☐ Itchy throat ☐ Wheezing ☐ Swelling of the lips or face
☐ Abdominal pain ☐ Throat tightness ☐ Change in voice
☐ Bluish color of lips and/or fingertips ☐ Other ________________________________

F. How many minutes after eating peanut or coming into contact with peanut did the reaction occur?

☐ Less than 5 minutes ☐ 5-10 minutes ☐ 10-20 minutes ☐ 20-30 minutes
☐ 30-45 minutes ☐ 45-60 minutes ☐ 1-2 hours ☐ I don't know
☐ If greater than 2 hours, when did the reaction occur?________________________________

G. How long did the symptoms last?

☐ Less than 1 hour ☐ Between 1 to 4 hours ☐ Between 4 to 8 hours
☐ Between 8 to 24 hours ☐ More than 24 hours ☐ I don't know

H. Was your child brought to a health care facility (hospital, clinic, CLSC, emergency room, etc…) for the treatment of the allergic reaction? ☐ Yes ☐ No ☐ I don't know
If **Yes**, please specify where: ____

**I.** What treatments were used to treat the reaction? **(Please specify for both categories)**	**Epipen®**	**Twinject™**	**Allerject™**	**Adrenaline/Epinephrine**	**Antihistamines (Benadryl®)**	**Ventolin® (Salbutamol)**	**Steroids (Prednisone)**	**Treated with medication name unknown**	**None**	**I don't know**
**Outside Health Care Facility (home, restaurant, school, etc…)**	☐	☐	☐		☐	☐	☐	☐	☐	☐
**At Health Care Facility**				☐	☐	☐	☐	☐	☐	☐

If **Other** please describe:

J. Was your child prescribed an epinephrine auto-injector for this reaction?
☐ No ☐ No, because my child already had an epinephrine auto-injector ☐ I don't know
☐ Yes, in the Emergency Room (ER)
☐ Yes, in another health care facility (please specify): ____

If **yes**, which type of epinephrine auto-injector was prescribed for this reaction?

☐ Epipen® ☐ Twinject™ ☐ Allerject™ ☐ Other, please specify: ______________ ☐ I don't know

K. What peanut-containing food or foods do you believe caused the reaction?

____

____

L. Please indicate the amount of food that was eaten before the reaction occurred? _______________________

____

10) <u>MOST SEVERE REACTION TO PEANUT</u> IN HIS/HER LIFE

- Was the first allergic reaction also the most severe one?
  - ☐ Yes Please go to **Question 11**.
  - ☐ No Please describe the most severe reaction below.

Date of reaction (Approximate month/year) ______________________________

A. Where did the reaction occur?
☐ Home ☐ Daycare-peanut allowed ☐ Daycare-peanut not allowed
☐ Restaurant ☐ School-peanut allowed ☐ School-peanut not allowed
☐ I don't know ☐Other _______________________

B. How did the reaction occur? ☐ After ingestion ☐ After skin contact ☐ I don't know
☐ After inhalation ☐ Not determined

C. Was the epinephrine auto-injector (Epipen®, Twinject™, Allerject™) available?
☐ Yes ☐ No ☐ I don't know

D. If an epinephrine auto-injector was used, was it his/her own? ☐ Yes ☐ No ☐ I don't know
If No, please specify whose epinephrine auto-injector was used: ____________________________________

E. Please check the symptoms that occurred:
☐ Hives ☐ Difficulty breathing ☐ Runny nose and watery eyes ☐ I don't know
☐ Redness ☐ Vomiting ☐ Passing out
☐ Itchy throat ☐ Wheezing ☐ Swelling of the lips or face
☐ Abdominal pain ☐ Throat tightness ☐ Change in voice
☐ Bluish color of lips and/or fingertips ☐ Other ____

F. How many minutes after eating peanut or coming into contact with peanut did the reaction occur?
☐ Less than 5 minutes ☐ 5-10 minutes ☐ 10-20 minutes ☐ 20-30 minutes
☐ 30-45 minutes ☐ 45-60 minutes ☐ 1-2 hours ☐ I don't know
☐ If greater than 2 hours, when did the reaction occur?____

G. How long did the symptoms last?
☐ Less than 1 hour ☐ Between 1 to 4 hours ☐ Between 4 to 8 hours
☐ Between 8 to 24 hours ☐ More than 24 hours ☐ I don't know

H. Was your child brought to a health care facility (hospital, clinic, CLSC, emergency room, etc…) for the treatment of the allergic reaction? ☐ Yes ☐ No ☐ I don't know

If **Yes**, please specify where: ____

**I.** What treatments were used to treat the reaction? **(Please specify for both categories)**	**Epipen®**	**Twinject™**	**Allerject™**	**Adrenaline/Epinephrine**	**Antihistamines (Benadryl®)**	**Ventolin® (Salbutamol)**	**Steroids (Prednisone)**	**Treated with medication name unknown**	**None**	**I don't know**
**Outside Health Care Facility (home, restaurant, school, etc…)**	☐	☐	☐		☐	☐	☐	☐	☐	☐
**At Health Care Facility**				☐	☐	☐	☐	☐	☐	☐

If **Other** please describe:

J. Was your child prescribed an epinephrine auto-injector for this reaction?

☐ No ☐ No, because my child already had an epinephrine auto-injector ☐ I don't know
☐ Yes, in the Emergency Room (ER)
☐ Yes, in another health care facility (please specify): ____________________________

If **yes**, which type of epinephrine auto-injector was prescribed for this reaction?
☐ Epipen® ☐ Twinject™ ☐ Allerject™ ☐ Other, please specify: ______________ ☐ I don't know

K. What peanut-containing food or foods do you believe caused the reaction?

____

____

L. Please indicate the amount of food that was eaten before the reaction occurred? ________________________

---

11) Has your child had any allergic reaction(s) to **peanut in the past year**?
☐ No ☐ Yes – Please tell us how many reactions in the past year: __________________

12) ACCIDENTAL REACTION TO PEANUT IN PAST YEAR

Date of **reaction to peanut** __________________ (Approximate month/year)

A. Where did the reaction occur?

☐ Home ☐ Daycare-peanut allowed ☐ Daycare-peanut not allowed
☐ Restaurant ☐ School-peanut allowed ☐ School-peanut not allowed
☐ I don't know ☐Other ____

B. How did the reaction occur? ☐ After ingestion ☐ After skin contact ☐ I don't know
☐ After inhalation ☐ Not determined

C. Was the epinephrine auto-injector (Epipen®,Twinject™) available? ☐ Yes ☐ No ☐ I don't know

D. If an epinephrine auto-injector was used, was it his/her **own**? ☐ Yes ☐ No ☐ I don't know

If **No**, please specify whose epinephrine auto-injector was used: ____________________________

E. Please check the symptoms that occurred:

☐ Hives ☐ Difficulty breathing ☐ Runny nose and watery eyes ☐ I don't know
☐ Redness ☐ Vomiting ☐ Passing out
☐ Itchy throat ☐ Wheezing ☐ Swelling of the lips or face
☐ Abdominal pain ☐ Throat tightness ☐ Change in voice
☐ Bluish color of lips and/or fingertips ☐ Other ____________________________________

F. How many minutes after eating peanuts or coming into contact with peanuts did the reaction occur?
☐ Less than 5 minutes ☐ 5-10 minutes ☐ 10-20 minutes ☐ 20-30 minutes
☐ 30-45 minutes ☐ 45-60 minutes ☐ 1-2 hours ☐ I don't know
☐ If greater than 2 hours, when did the reaction occur? __________________________________

G. How long did the symptoms last?

☐ Less than 1 hour ☐ Between 1 to 4 hours ☐ Between 4 to 8 hours
☐ Between 8 to 24 hours ☐ More than 24 hours ☐ I don't know

H. Was your child brought to a health care facility (hospital, clinic, CLSC, emergency room, etc…) for the treatment of the allergic reaction? ☐ Yes ☐ No ☐ I don't know
If **Yes**, please specify where: ____.

I. What treatments were used to treat the reaction? **(Please specify for both categories)**

	**At Health Care Facility**	**Outside Health Care Facility** (home, restaurant, school, etc…)
None	☐	☐
Epipen®, Twinject™		☐
Adrenaline/Epinephrine	☐	
Antihistamines (Benadryl®, Atarax®)	☐	☐
Ventolin® (Salbutamol)	☐	☐
Steroids (Prednisone)	☐	☐
Treated with medication name unknown	☐	☐
I don't know	☐	☐

Other ____

J. Was your child prescribed an epinephrine auto-injector for this reaction?
☐ No, because the child already had and epinephrine auto-injector
☐ Yes, in the Emergency Room (ER)
☐ Yes, in another health care facility (please specify) _________________________________
☐ No
☐ I don't know

K. What peanut-containing food or foods do you believe caused the reaction?

____
____

L. Please indicate the amount of food that was eaten before the reaction occurred?

____
____

SOCIAL INFORMATION

13) Age of child's mother/female guardian: _________

14) Age of child's father/male guardian: __________

15) Education of child's mother/female guardian:
- ☐ High School not completed
- ☐ High School completed
- ☐ CEGEP/College
- ☐ University

16) Education of child's father/male guardian:
- ☐ High School not completed
- ☐ High School completed
- ☐ CEGEP/College
- ☐ University

17) What is your child's cultural background? **Categories taken from Census Canada 2001*

☐ White ☐ Chinese ☐ Japanese ☐ Korean
☐ Black ☐ Filipino ☐ Arab ☐ Latin American
☐ South Asian (e.g. East Indian, Pakistani, Sri Lankan, etc.)
☐ Southeast Asian (e.g. Cambodian, Indonesian, Laotian, Vietnamese, etc)
☐ West Asian (Afghan, Iranian, etc)
☐ Aboriginal (North American Indian, Métis, Inuit)
☐ Other: ____

Thank you for completing this questionnaire!
