# Supplementary material for: Accidental exposures to peanut in a large cohort of Canadian children with peanut allergy
Source: Clin Transl Allergy. 2015 Apr 2;5:16. doi: 10.1186/s13601-015-0055-x (PMC4389801; doi:10.1186/s13601-015-0055-x)
Supplement: Additional file 2: — Peanut allergy registry – follow up. Follow up questionnaire sent to patients participating in the peanut allergy registry. [file 13601_2015_55_MOESM2_ESM.pdf]

Peanut Allergy Registry – Follow Up

Date: (dd/mmm/yyyy)______________________________ **Study ID #:** ☐ ☐☐☐☐

TO BE COMPLETED BY PARENT/GUARDIAN OF PEANUT ALLERGIC PARTICIPANT

1) Has your child developed any of the following conditions since you responded to the previous questionnaire?

☐ Asthma
☐ Eczema
☐ Hay fever or allergic rhinitis (stuffy nose or frequent sneezing)
☐ Hives (itchy skin rash that comes and goes over minutes to hours)
☐ Anaphylactic reaction such as wheezing or other breathing difficulties, total body hives, vomiting or loss of consciousness
☐ Food allergy (list foods) ____

2) Please check all that apply to **your child's sibling(s):**

☐ My child does not have any siblings (Please go to question 4)

Asthma
Your child's sibling(s) ☐ Yes ☐ No ☐ Don't know ☐ Resolved

Eczema
Your child's sibling(s) ☐ Yes ☐ No ☐ Don't know ☐ Resolved

Hay fever or allergic rhinitis (stuffy nose or frequent sneezing)
Your child's sibling(s) ☐ Yes ☐ No ☐ Don't know ☐ Resolved

Hives (itchy skin rash that comes and goes over minutes to hours)
Your child's sibling(s) ☐ Yes ☐ No ☐ Don't know ☐ Resolved

Anaphylaxis (wheezing or other breathing difficulties, total body hives, vomiting or loss of consciousness from an allergic reaction)
Your child's sibling(s) ☐ Yes ☐ No ☐ Don't know ☐ Resolved

3) Do your child's sibling(s) have any food allergies?

List food (s) ____

4) Which Epinephrine auto-injector (s) does your child **currently have**?

☐ Epipen® ☐ Twinject™ ☐ Allerject™ ☐ Other, please specify: _______________
☐ I don't know ☐ My child does not have an Auto-Injector

5) Has your child had any allergic reaction(s) to **peanut in the past year**?

☐ No ☐ Yes – Please tell us how many reactions in the past year: _______

6) ACCIDENTAL REACTION TO PEANUT IN PAST YEAR

Date of **reaction to peanut** ___________________ (Approximate month/year)

A. Where did the reaction occur?

☐ Home ☐ Daycare-peanut allowed ☐ Daycare-peanut not allowed
☐ Restaurant ☐ School-peanut allowed ☐ School-peanut not allowed
☐ I don't know ☐Other ____

B. How did the reaction occur? ☐ After ingestion ☐ After skin contact ☐ I don't know
☐ After inhalation ☐ Not determined

C. Was the epinephrine auto-injector (Epipen®,Twinject™) available? ☐ Yes ☐ No ☐ I don't know

D. If an epinephrine auto-injector was used, was it his/her **own**? ☐ Yes ☐ No ☐ I don't know
If **No**, please specify whose epinephrine auto-injector was used: ____________________________

E. Please check the symptoms that occurred:

☐ Hives	☐ Difficulty breathing	☐ Runny nose and watery eyes	☐ I don't know
☐ Redness	☐ Vomiting	☐ Passing out	
☐ Itchy throat	☐ Wheezing	☐ Swelling of the lips or face	
☐ Abdominal pain	☐ Throat tightness	☐ Change in voice	

☐ Bluish color of lips and/or fingertips ☐ Other ____________________________________

F. How many minutes after eating peanuts or coming into contact with peanuts did the reaction occur?

☐ Less than 5 minutes	☐ 5-10 minutes	☐ 10-20 minutes	☐ 20-30 minutes
☐ 30-45 minutes	☐ 45-60 minutes	☐ 1-2 hours	☐ I don't know

☐ If greater than 2 hours, when did the reaction occur? ___________________________________

G. How long did the symptoms last?

☐ Less than 1 hour	☐ Between 1 to 4 hours	☐ Between 4 to 8 hours
☐ Between 8 to 24 hours	☐ More than 24 hours	☐ I don't know

H. Was your child brought to a health care facility (hospital, clinic, CLSC, emergency room, etc…) for the treatment of the allergic reaction? ☐ Yes ☐ No ☐ I don't know

If **Yes**, please specify where: ____

I. What treatments were used to treat the reaction? **(Please specify for both categories)**

	**At Health Care Facility**	**Outside Health Care Facility** (home, restaurant, school, etc…)
None	☐	☐
Epipen®, Twinject™		☐
Adrenaline/Epinephrine	☐	
Antihistamines (Benadryl®, Atarax®)	☐	☐
Ventolin® (Salbutamol)	☐	☐
Steroids (Prednisone)	☐	☐
Treated with medication name unknown	☐	☐
I don't know	☐	☐

Other ____

J. Was your child prescribed an epinephrine auto-injector for this reaction?

☐ No, because the child already had and epinephrine auto-injector
☐ Yes, in the Emergency Room (ER)
☐ Yes, in another health care facility (please specify) ___________________________________
☐ No
☐ I don't know

K. What peanut-containing food or foods do you believe caused the reaction?

____

____

L. Please indicate the amount of food that was eaten before the reaction occurred?

____

____

7) Do you consider your child to still be **peanut** allergic? ☐ Yes ☐ No **If No,** is it due to (please check all answers that apply):

☐ My child has eaten peanut two or more times in the past year and has **not** had a reaction

☐ My child has been tested by an allergist and found to no longer be peanut allergic
If your child was tested by an allergist and found to no longer be peanut allergic please tell us the date of the test: (mm/yyyy) __________________________

Thank you for completing this questionnaire
